# Editorial: Bedside visual image technologies for respiratory and circulatory management in intensive care settings

**DOI:** 10.3389/fmed.2022.973679

**Published:** 2022-07-18

**Authors:** Huaiwu He, Yun Long, Knut Möller, Zhanqi Zhao

**Affiliations:** ^1^State Key Laboratory of Complex Severe and Rare Disease, Department of Critical Care Medicine, Peking Union Medical College Hospital, Peking Union Medical College, Chinese Academy of Medical Sciences, Beijing, China; ^2^Institute of Technical Medicine, Furtwangen University, Villingen-Schwenningen, Germany; ^3^Department of Biomedical Engineering, Fourth Military Medical University, Xi'an, China

**Keywords:** critical EIT, critical ultrasound, lung ventilation, lung perfusion, respiratory and circulatory management

Cardiovascular and pulmonary systems are the major systems that suffer from critical diseases and injuries among patients. Accessible and accurate tools providing diagnosis and monitoring information are warranted to assist individualized therapy and decision making. Up-to-date, novel imaging techniques emerge thanks to the advancement in the field, including novel imaging modalities that have not been widely used in clinical practice. Bedside visual image technologies, including electrical impedance tomography (EIT) for ventilation and perfusion, and critical care ultrasound for alveolar atelectasis and cardiac output assessments, have gained great attentions in ICU/operating theater. In the current Research Topic, we are happy to collect one review, two case reports, one simulation, and five clinical studies investigated in more than 250 subjects to update our knowledge in the field.

Interactions among cardiovascular, pulmonary and other systems are often complicated. Many diseases could lead to multiple-organ failure. One example is hepatopulmonary syndrome, which causes pulmonary vascular dysfunction secondary to liver cirrhosis (1). Evidence of intrapulmonary vascular dilatation is one of the diagnostic criteria for hepatopulmonary syndrome. Therefore, imaging examination could be one of the critical measures to identify pulmonary vasodilatation. In the current Research Topic, Luo and Du reviewed the recent advance diagnostic imaging techniques for hepatopulmonary syndrome, including ultrasound, dynamic pulmonary perfusion imaging, pulmonary angiography, and computed tomography. They discussed the pros and cons of the current techniques and pointed out the need and room for further development of imaging techniques.

In addition to the multiple-organ interactions, treatment of cardiopulmonary diseases may introduce some psychological impairments (2). Some studies indicated that revisiting the ICU treatment period could be helpful *via* providing patients with necessary information (3). Vlake et al. demonstrated in a patient recovered from COVID-19 that virtual reality intervention provided a valuable adjunct to improve patient's psychological status. The development and application of the virtual reality module require an interdisciplinary cooperation. To increase the effectiveness, the module might need to be constructed individually to meet the experience of the patient groups. Therefore, it is a promising field that requires further investigation.

The rest of the papers in the Research Topic are either ultrasound or EIT related, which implicates rapid development in these two imaging modalities. The use of medical ultrasound for cardiovascular system can date back to 1960s (4). To date, ultrasound is used as a diagnostic tool as well as guiding tool for certain maneuvers. To evaluate the right ventricle filling state, Zhao et al. used echocardiography to dynamically monitor central venous pressure and the size of right ventricle. They were able to clarify the correlation between left ventricle stroke volume and negative fluid balance in 71 patients with elevated central venous pressure *via* comparing hemodynamic and echo parameters at baseline and after negative fluid balance. Ultrasound-guided catheterization has been used for a decade but the success rate may depend on arterial depth (5). Tian et al. retrospectively analyzed 119 patients and demonstrated a potential correlation between first-attempt success and arterial depth.

EIT is a novel non-invasive bedside imaging modality (6). EIT can be used to monitor the ventilation distributions during the entire ventilation support period. A recently important milestone is the evidence from two randomized-controlled trials that EIT-guided positive end-expiratory pressure (PEEP) settings may improve outcomes (length of hospital stay, mortality etc.) in patients with acute respiratory distress syndrome (ARDS) (7, 8). Li et al. evaluated the possibility to predict weaning outcome in patients with delayed upper abdominal surgery. Another team tested the ability of EIT to predict outcome of high flow nasal cannula therapy. These studies broadened the use of EIT to support the respiratory management in ICU. Lung perfusion assessed with EIT helps physicians to understand the ventilation-perfusion matching status and identify the potential reasons for respiratory failure (9). Hypertonic saline bolus is injected *via* central venous catheter to augment the impedance signal. Wang et al. shared their experience in managing a patient with high-risk pulmonary embolism. Not only adult patients can benefit from EIT. Ren et al. compared PEEP titration with global and regional compliance calculated with EIT data in pediatric ARDS. We believe more and more EIT applications in pediatric patients may be published when the device manufactures provide EIT for such patient group. Various requirements need to be considered for different applications and different patient groups, such as electrode size, position, noise suppression, baseline drift and moving artifacts. Yang et al. presented mathematical method to overcome some of the issues during practical EIT measurements. Although the effectiveness was only validated in limited patient data but the results were promising.

Although medical ultrasound has been reported since several decades, due to the lack of bedside tools, lung ultrasound became one of the hot topics in recent years (10, 11). Lung and heart ultrasound provides different information compared to EIT: ultrasound could provide morphology information of cardiopulmonary lesions, cardiac systolic and diastolic function, special signs of etiologies, whereas EIT could make a rapid functional assessment of regional lung ventilation, perfusion and the corresponding matching. Development of personalized medicine and clinical decision may benefit from the information of both bedside techniques. In clinical practice, we propose to combine these two techniques to manage patients with cardiopulmonary dysfunctions (Figure 1). By combining EIT and ultrasound, we can make a holistic management for the circulatory and respiratory failure. Further studies are warranted to examine whether the proposed workflow can improve patient outcomes.

**Figure 1:**
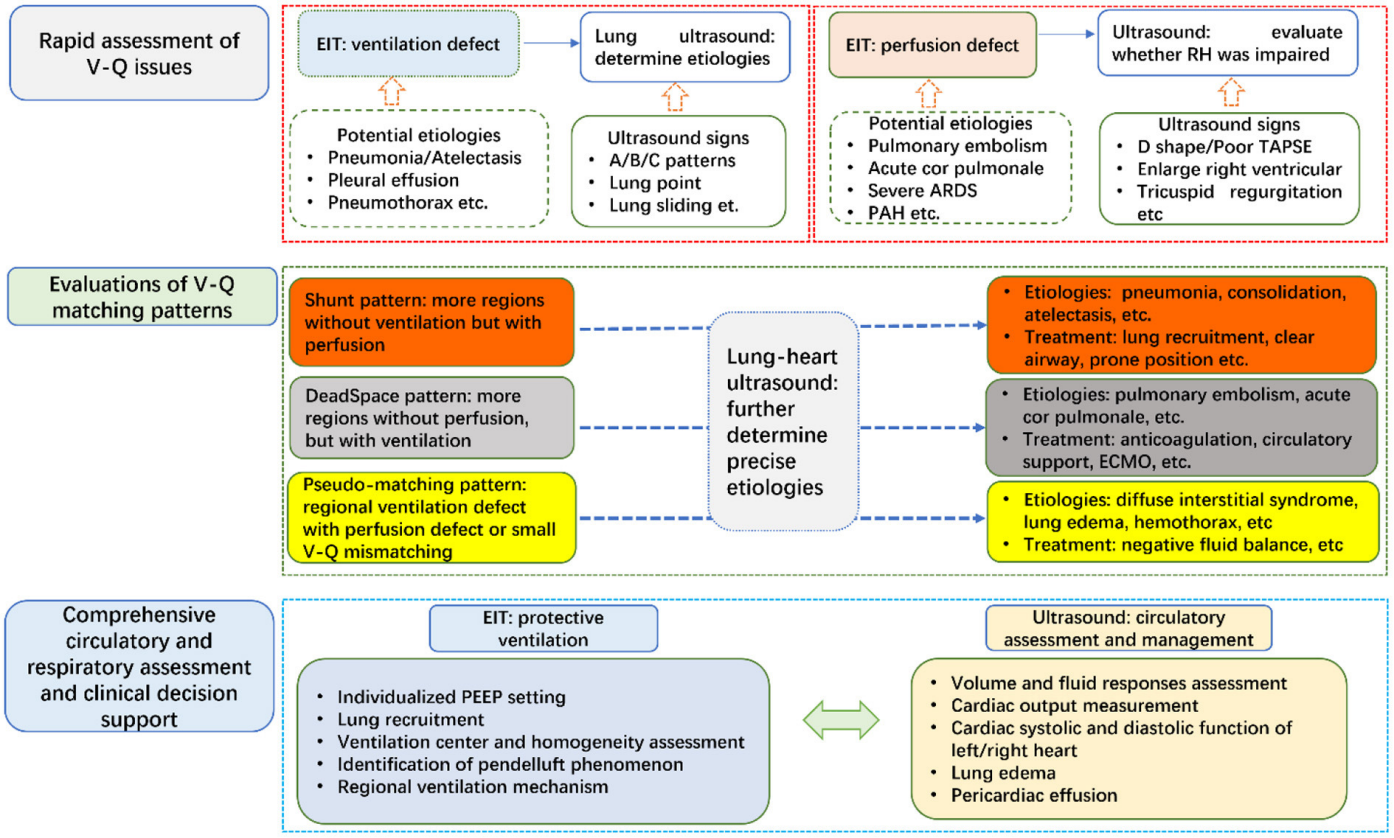
Combination of chest EIT and lung ultrasound for respiratory and circulatory managements in ICU. TAPSE, Tricuspid annular plane systolic excursion; PAH, pulmonary arterial hypertension; ARDS, acute respiratory distress syndrome; V-Q, ventilation-perfusion; PEEP, positive end-expiratory pressure; ECMO, extracorporeal membrane oxygenation.

## Author contributions

HH and ZZ drafted the manuscript. YL and KM critically revised the manuscript. All authors have approved the final version.

## Conflict of interest

The authors declare that the research was conducted in the absence of any commercial or financial relationships that could be construed as a potential conflict of interest.

## Publisher's note

All claims expressed in this article are solely those of the authors and do not necessarily represent those of their affiliated organizations, or those of the publisher, the editors and the reviewers. Any product that may be evaluated in this article, or claim that may be made by its manufacturer, is not guaranteed or endorsed by the publisher.
